# The aftermath of war; mental health, substance use and their correlates with social support and resilience among adolescents in a post-conflict region of Sri Lanka

**DOI:** 10.1186/s13034-023-00648-1

**Published:** 2023-08-31

**Authors:** Lasith Dissanayake, Sameeha Jabir, Thomas Shepherd, Toby Helliwell, Lavan Selvaratnam, Kaushalya Jayaweera, Nihal Abeysinghe, Christian Mallen, Athula Sumathipala

**Affiliations:** 1 Institute for Research and Development in Health & Social Care, Off Robert Gunawardena Mawatha, Battaramulla, 10120 Sri Lanka; 2 School of Medicine, Keele University, Staffordshire, ST5 5BG United Kingdom; 3 Ministry of Health and Indigenous Medical Services, Colombo, 01 Sri Lanka

**Keywords:** Adolescents, Substance use, School Dropouts, Depression, Hopelessness, Sri Lanka

## Abstract

**Background:**

Armed conflicts impact on the health and well-being of everyone, but its effect on adolescent mental health is a significant, yet under-explored area in global health. Mental health disorders which develop during adolescence often lead to behavioural problems, risky decision-making, under-age substance use and can adversely impact on educational attainment. This study aimed to estimate the prevalence of common mental disorders, substance use and their correlates with social support and resilience among adolescents (age 12–19 years) in Vavuniya; a post-conflict region of Sri Lanka.

**Methods:**

A population-based cross-sectional study was conducted, with a modified cluster sampling method used for participant selection. Eight culturally adapted instruments were used for data collection. A total of 585 adolescents participated in the study. Analyses were performed using SPSS Version 23 statistical software package. All statistical tests were two-sided (p < 0.05) and p-values less than 0.05 were considered significant. Chi-square tests were used to explore associations between variables of interest. Spearman rank order correlation was used to examine correlations among depression, hopelessness, quality of life, social support, and resilience.

**Results:**

The mean age of participants was 15.02 (± 2.13) years. Ninety-one (15.6%) participants reported being exposed to one or more war-related events, and 85 (93.4%) participants in this group reported being internally displaced due to war. Fifty-two (8.9%) had dropped out of school and the prevalence of depression (3.9%) and substance use (7%) were low. Correlational analyses revealed that depression and hopelessness were significantly negatively correlated with social support, resilience, and quality of life (p < 0.01). Linear regression analysis suggested that 40% of the variance in resilience of the participants can be explained by perceived social support.

**Conclusion:**

The low prevalence of hopelessness and depression highlights the resilience of this group in the face of adversity. Furthermore, significant negative correlations between hopelessness and depression with perceived social support and resilience suggest that social support and resilience could be protective factors against mental health issues in these adolescents. However, the prevalence of school dropouts calls for a focus on academic attainment to promote better educational outcomes in the adolescents of this conflict-affected region.

## Introduction

### Adolescent Mental Health

Armed conflicts are known to have a marked impact on the health and well-being of children and adolescents who may experience an array of negative events including displacement, separation/death of parents, witnessing death and destruction, and being forced to leave school [1]. Whilst some may adjust to the trauma and recover from the emotional distress, the direct or indirect exposure to war can inflict harm on adolescents that persists over their entire life course. The World Health Organisation (WHO) estimates that “among people who have experienced war or other conflicts in the previous 10 years, one in five (22%) will have depression, anxiety, post-traumatic stress disorder, bipolar disorder or schizophrenia” [2, 3].

The impact on adolescent mental health is one of the most significant, yet under-explored after-effects of war [4, 5]. The long-term accumulation of a range of stressors and risk factors puts children and adolescents in post-conflict regions at an increased risk of mental health problems such as depression and anxiety [6]. The four “P” framework of case formulation (predisposing, precipitating, perpetuating, and protective factors) also provide insight into the factors that may contribute to the development of mental health disorders in this group [7, 8]. Mental health disorders in adolescents are also known to present themselves in other ways such as through absenteeism, aggressiveness, substance use, medically unexplained pain, and antisocial behaviour, especially among those in post-conflict regions [9, 10].

### Impact of Civil War in Sri Lanka

The three decade long civil war (1983–2009) in Sri Lanka is undeniably the most brutal internal conflict which befell the country. It took the lives of more than 70,000 people and left nearly a million people in the Northern and Eastern provinces displaced. Furthermore affected regions were left with weakened infrastructure, increased poverty and dramatically under-functioning education and healthcare systems [11, 12]. This civil conflict also had a devastating impact on the mental health of the country’s population, especially on children and adolescents in the areas of Northern and Eastern provinces [9, 13].

The lack of infrastructure, along with under-functioning healthcare systems in post-conflict settings in the country, in addition to the social stigma attached with mental health problems, poses a serious risk to adolescent mental health [3, 14]. Moreover, despite having an effective primary health care system, Sri Lanka has a significant mental health treatment gap, particularly in post-conflict areas [9, 15].

Vavuniya district is in the Northern Province of the country and experienced high levels of conflict during the civil war. It was also one of the districts that experienced repeated displacements during the conflict, resulting in 256,287 internally displaced persons (IDPs) moving to Vavuniya during the civil war period [16].

### Objectives

The study aimed to estimate the prevalence of common mental disorders, suicidal ideation, substance use (alcohol, tobacco, and illicit drugs) and their correlates with social support and resilience among adolescents in Vavuniya, a post-conflict region of Sri Lanka.

### Methodology

#### Study Design

This cross-sectional study used a population-based sample in Vavuniya district of Sri Lanka. Data was collected from July 2019 to October 2020.

### Study setting

Vavuniya district is one of the 25 districts of Sri Lanka, and it can be divided in to four Divisional Secretariat Divisions (DSDs) and 102 Grama Niladhari Divisions (GNDs) (the smallest administrative unit) [17]. The population in Vavuniya district is multi-ethnic and multi-cultural with 82.37% of Sri Lankan Tamil ethnicity [18]. The district has both rural and urban sectors, with the majority of the population (79.8%), residing in rural areas. Vavuniya DSD is the most populated DSD and is home to 117,153 people [18].

### Study Population

The study population included adolescents aged 12 to 19 years living permanently in Vavuniya district for five or more years. The initial aim was to recruit adolescents aged between 10 and 19 years. However, following Community Involvement and Engagement (CIE) activities and the recommendations of the Ethics Review Committee (ERC), this was changed to 12 years. A five-year residency period was implemented, as it takes time to become familiarized with Vavuniya.

#### Inclusion criteria

Adolescents who speak Tamil, Sinhala or English languages and living permanently in the district of Vavuniya for five or more years were included in this study.

#### Exclusion criteria

Adolescents were excluded if they had a diagnosis of severe hearing problems, speech problems, autism and significant intellectual disability, severe chronic diseases such as cancer or significant respiratory problems. Adolescents living in non-household arrangements, such as orphanages, and those who were unable to understand the process of consent, or the questionnaires were also excluded.

### Sample size calculation

Vavuniya district has an adolescent population (ages 10 to 19 years) of 34,123 [18]. However, exact prevalence estimates for mental disorders and substance use among adolescents were not available for the district, therefore; an estimated prevalence of 50% was used [19]. OpenEpi, Version 3, open-source calculator was used to calculate the sample size (http://openepi.com/) [20].

n = [(DEFF) Np(1 - p)]/[(d^2^/Z^2^(1 - α/2))(N − 1) + p(1 - p)]

An initial sample size of 570 was obtained for high precision (95% confidence intervals) and a design effect of 1.5. Assuming a non-response rate of 20% the final sample size was inflated to 700 participants.

### Sampling and participant recruitment

A cluster sampling method was used to select the sample. A modified version of the ‘Expanded Program on Immunization Cluster Survey design’ widely used by the WHO [21], which has been demonstrated to improve on the original design [22]. This approach is widely used in LMIC settings and is a practical method to obtain a representative sample for a cross-sectional study in a large geographical area.

The total sample originated from 29 GNDs, with 24 participants from 28 GNDs and 48 participants from one GND. We selected the eldest adolescent, if two or more eligible adolescents were living in one housing unit.

### Instruments

Data was collected using the following instruments.

- Socio-demographic Questionnaire.
- Youth Quality of Life Questionnaire [23].
- Child and Youth Resilience Measure [24].
- Multidimensional Scale of Perceived Social Support [25].
- Patient Health Questionnaire [26, 27].
- Brief Questionnaire on War [28].
- Beck Hopelessness Scale [29].
- Questionnaire on Substance use.

A comprehensive substance use questionnaire was developed and adapted for this study based on the Student Questionnaire on Substance Use developed by the United Nations Office on Drugs and Crime [30].

### Culturally adapting the Instruments

The languages used in Vavuniya are primarily Tamil & Sinhala and therefore, study instruments in the English language were translated and adapted to these two languages. Rather than following the traditional forward and backwards direct translation method, we used a qualitative and quantitative approach with consensus generation developed by Sumathipala and Murray in 2000, which is widely used internationally and recommended locally [31–33].

Two teams with three and five members per group, were involved in translating the documents to Sinhala and Tamil languages, respectively. Due to the number of different dialects used in the Vavuniya region we formed a CIE group consisting of lay people, counsellors, a teacher, and a pastor. Four members of the nine-member bilingual CIE group had adolescent children. The CIE group worked with the study team, checking for errors in translation, sentence composition, and the meaning of translated phrases in the context of Vavuniya. Words and phrases were changed where necessary to match the colloquial language of Vavuniya while retaining the original theme. Based on the CIE group’s advice, questionnaires deemed to be too sensitive for children were removed; especially a questionnaire which focused on suicidal ideation.

### Ethical considerations and data Collection Method

The ERC suggested to exclude questions on suicidal ideation and hopelessness, and to increase the minimum age of the recruitment criteria up to 12 years which we adopted.

Research Assistants (RAs) received training on the ethical complications and sensitivity related to research on adolescents. First, they explained the study to the parents/guardians of the adolescent, and obtained their permission to speak with the adolescent. After which the study was explained to the adolescents in simple language. Information leaflets were given to both parents/guardians and the adolescents. Written; permission from parents, and assent/consent from adolescents was obtained before data collection.

To protect participant privacy the interviews were conducted out of their houses and in an open area, with the permission of parents/guardians. Printed questionnaire booklets were used, and the data collection was conducted using a combination of interviewer administered and self-administered methods. Sensitive questions on depression and certain sections of the substance use questionnaire were filled out by the participants themselves.

### Statistical analysis

Analyses were performed using SPSS Version 23 statistical software package. All statistical tests were two-sided (*p* < 0.05) and p-values less than 0.05 were considered significant. Chi-square tests were used to explore the association between variables of interest. Spearman rank order correlation was used to examine correlations among depression, hopelessness, quality of life, social support, and resilience. Linear regression analysis was used to explore the independent effect of different variables on depression and hopelessness symptoms.

## Results

### Demographic characteristics

Due to the COVID-19 pandemic and restrictions that were in place at the time, data collection stopped in October 2020. A total of 585 adolescents participated in the study. The mean age of participants was 15.02 (± 2.13) years. There were almost equal representations of males and females in the study, with 50.4% of the participants being female. Among the participants, 52 (8.9%) had dropped out of school. Out of those who had dropped out of school, a higher percentage were males (n = 29; 55.8%), than females (n = 23; 44.2%).

**Table 1:** Distribution of respondents by socio-demographic characteristics

Variables	Males		Females		Both Sexes	
Age group (In years)	n	%	n	%	n	%
12–14 years	134	46.2	113	38.3	247	42.2
15–16 years	75	25.9	100	33.9	175	29.9
17–19 years	81	27.9	82	27.8	163	27.9
	**n = 290**		**n = 295**		**n = 585**	
**Ethnicity**						
Tamil	264	91.0	273	92.5	537	91.8
Sinhala	26	9.0	22	7.5	48	8.2
	**n = 290**		**n = 295**		**n = 585**	
**District Secretariat Division**						
Vavuniya North	22	7.6	26	8.8	48	8.2
Vavuniya South	26	9.0	22	7.5	48	8.2
Vavuniya	196	67.6	197	66.8	393	67.2
Vengalacheddikulam	46	15.9	50	16.9	96	16.4
	**n = 290**		**n = 295**		**n = 585**	
**Dropped out of school**						
Yes	29	10.0	23	7.8	52	8.9
No	261	90.0	272	92.2	533	91.1
	**n = 290**		**n = 295**		**n = 585**	
**Mother’s availability at home**						
Yes	261	90.0	267	90.5	528	90.3
No	29	10.0	28	9.5	57	9.7
	**n = 290**		**n = 295**		**n = 585**	
**Father’s availability at home**						
Yes	224	77.2	216	73.2	440	75.2
No	66	22.8	78	26.4	144	24.6
	**n = 290**		**n = 294**		**n = 584**	

### War-related events

Among the 585 respondents, 91 (15.6%) reported being exposed to one or more war-related events. A majority (n = 85, 93.4%) of the participants who had been exposed to war-related events reported that they were internally displaced due to the war. Families of 80 (87.9%) participants had lost property and other belongings due to conflicts in the region.

**Table 2:** Distribution of respondents by exposure to different war-related events

Variables	Males		Females		Both Sexes	
Exposure to at least one war-related event	n	%	n	%	n	%
Yes	39	13.4	52	17.6	91	15.6
No	251	86.6	243	82.4	494	84.4
	**n = 290**		**n = 295**		**n = 585**	
**Sustained injuries because of war**						
Yes	0	0	2	0.7	2	0.3
No	290	100%	293	99.3	583	99.7
	**n = 290**		**n = 295**		**n = 585**	
**Lost a family member / close friend because of war**						
Yes	20	6.9	22	7.5	42	7.2
No	270	93.1	273	92.5	543	92.8
	**n = 290**		**n = 295**		**n = 585**	
** A family member or friend was injured because of war**						
Yes	18	6.2	20	6.8	38	6.5
No	272	93.8	275	93.2	547	93.5
	**n = 290**		**n = 295**		**n = 585**	
**Displaced because of war**						
Yes	35	12.1	50	16.9	85	14.5
No	255	87.9	245	83.1	500	85.5
	**n = 290**		**n = 295**		**n = 585**	
**Lost property because of war**						
Yes	32	11.0	48	16.3	80	13.7
No	258	89.0	247	83.7	505	86.3
	**n = 290**		**n = 295**		**n = 585**	

### Substance use

Respondents were asked about their tobacco, alcohol and illegal substance use with 7.0% (n = 41) reporting a history of using these substances during their lifetime. The majority of the participants (females − 98.6% and males − 87.2%) were lifetime abstainers of alcohol, tobacco, and other substances. Among the males who reported tobacco, alcohol or any substance use, the majority were in the age group of 15–19 years (81.1%).

**Table 3:** Lifetime alcohol, tobacco, and substance use

Variables	Males		Females		Both Sexes	
Exposure to lifetime alcohol, tobacco, or substance	n	%	n	%	n	%
**Yes**	37	12.8	4	1.4	41	7.0
**No**	253	87.2	291	98.6	544	93.0

Among participants who reported lifetime tobacco, alcohol or any substance use, the majority (n = 33, 80.5%) reported using tobacco-containing products. Most (34.21%) of the respondents reported that the first substance they used was Bidi (a tobacco-based small cigarette), followed by Betel quid (23.7%) and Beer (15.8%). The rest (26.29%) reported using other substances such as Cigarettes, Spirits, Wine, Babul (pieces of Babul plant with tobacco) and Mawa (dried areca nut with tobacco flakes).

Curiosity (80%), a friend/friends using it (72.5%) and easy access to a particular substance (70%) were the reasons reported by adolescents for substance use. Some participants also reported that they used substances because they were feeling unhappy or lonely (27.5%).

### Mental Health Disorders

The study explored different mental health issues and symptoms including depression and hopelessness. Moderate - severe depression was present in only 3.9% (n = 23) of the participants. Moderate to severe hopelessness was reported by 5.2% (n = 8) of the participants aged 17 years and above.

**Table 4:** Prevalence of Depression and Hopelessness in the study population

Variables	Males		Females		Both Sexes	
Depression	n	%	n	%	n	%
**Minimal – mild depression**	279	96.2	283	95.9	562	96.1
**Moderate – moderately severe depression**	11	3.8	12	4.1	23	3.9
	**n = 290**		**n = 295**		**n = 585**	
**Hopelessness**	**n**	**%**	**n**	**%**	**n**	**%**
**None to mild hopelessness**	73	97.3	74	92.5	147	94.8
**Moderate to severe hopelessness**	2	2.7	6	7.5	8	5.2
	**n = 75**		**n = 80**		**n = 155**	

### Quality of life, perceived social support and resilience

The study assessed quality of life, perceived social support and resilience. Respondents reported that they received high levels of social support with a mean score of 6.24 ± 0.80 (the maximum score being 7.0).

**Table 5:** Perceived Social Support

Variables	Males		Females		Both Sexes	
Perceived social support	n	%	n	%	n	%
**Low social support**	0	0	1	0.4	1	0.2
**Moderate social support**	25	10.3	16	6.5	41	8.4
**High social support**	217	89.7	228	93.1	445	91.4

The mean quality of life score was 88.4 (± 8.73) (the maximum score − 100). Participants reported a mean resilience score of 54.10 (± 5.15) (the maximum score − 60).

Depression, substance use and dropping out of school were prevalent in the age 15–19 years category. Depression was significantly associated with hopelessness, age, dropping out of school and perceived social support. Hopelessness showed a significant association with perceived social support. The chi-square associations are shown in Table 6.

**Table 6:** Association of Depression and Hopelessness with selected variables

	Variables	Chi-square value	P-value
**Depression**	Hopelessness	10.458	0.01
	Substance use	1.338	0.21
	Age	7.040	0.01
	Gender	0.029	0.86
	Perceived social support	11.081	0.01
	Dropping out of school	8.744	0.01
**Hopelessness**	Substance use	1.273	0.26
	Gender	1.847	0.28
	Perceived social support	10.031	0.01
	Dropping out of school	0.910	0.68

The crude odds ratio of depression to hopelessness was 9.20 (95% CI: 1.89–44.77). High perceived social support was seen to reduce the odds of both depression (OR: 0.21; 95% CI: 0.08–0.57) and hopelessness (OR: 0.13; 95% CI: 0.03–0.55).

**Table 7:** Strength of Association of Depression and Hopelessness with selected variables

	Variables	Unadjusted Odds	P-value
**Depression**	Hopelessness	9.200	< 0.001
	Substance use	2.068	0.25
	Age	2.969	0.01
	Gender	1.075	0.86
	Perceived social support	0.209	< 0.001
	Dropping out of school	3.959	0.01
**Hopelessness**	Substance use	2.549	0.27
	Gender	2.959	0.19
	Perceived social support	0.125	0.01
	Dropping out of school	0.369	0.36

#### Correlational analysis and linear regression

Bivariate Spearman correlations suggested that depression and hopelessness had highly negative correlations with social support and quality of life. Perceived social support was negatively correlated with depression (r=-0.20, p < 0.01 and hopelessness (r=-0.40, p = 0.000). Quality of life was also negatively correlated with depression (r=-0.28, p = 0.000) and hopelessness (r=-0.40, p < 0.01), and positively related with social support (r = 0.50, p = 0.000). Resilience was negatively correlated with hopelessness (r=-0.49, p < 0.01) and depression (r= -0.14, p < 0.01), and positively correlated with perceived social support (r = 0.60, p < 0.01).

Linear regression analysis revealed that the R^2^ value between resilience and perceived social support was 0.40, suggesting that 40% of the variance in resilience of the participants can be explained by perceived social support. Also, the β coefficient was 4.04 (CI: 3.55–4.533), meaning that for a unit increase in perceived social support scores, resilience scores increase by 4.04.

The R^2^ value between youth quality of life and perceived social support was 0.22, suggesting that 22% of the variance in the quality of life of the participants can be explained by perceived social support. The β coefficient was 5.6 (CI: 4.650–6.627), meaning that for a unit increase in perceived social support scores, resilience scores increase by 5.6.

## Discussion

Empirical research evidence suggests that violence and stress related to war can have a detrimental impact on children’s mental health [34, 35]. A study conducted among 551 adolescents in Northern Uganda, four years after the civil war ended reported that depressive symptoms were positively associated with multiple risk behaviours (OR: 1.11; 95% CI: 1.07–1.14, p < 0.001) [36]. A similar study conducted with 2766 adolescents in Donetsk, Ukraine two years after the Russian invasion in 2014, reported significantly increased risks of PTSD (OR: 4.11; 95% CI: 2.37–7.13), severe anxiety (OR: 3.10; 95% CI: 1.83–5.27), and moderately severe/severe depression (OR: 2.65; 95% CI: 1.79–3.92) [37]. The present study was conducted in the Vavuniya district, ten years after the civil war in Sri Lanka ended in 2009. The oldest adolescents (19 years of age) who participated in the study were 9-year-old children during the last year of the civil war. There is a high possibility that the participants might have already recovered from the trauma and were leading normal lives, and this could be one of the main reasons for the significantly low prevalence of depression (3.9%) reported among adolescents.

Another significant finding was the low prevalence of hopelessness (5.2%) among the participants. Participants reported high resilience scores (Mean = 54.10, the maximum score − 60) and high perceived social support scores (Mean = 6.28, the maximum score − 7) despite Vavuniya being a conflict-affected region. Furthermore, hopelessness and depression were significantly negatively correlated with perceived social support and resilience (P < 0.05), suggesting that social support and resilience could be protective factors against mental health issues in these adolescents.

According to Somasundaram (2010), some, if not, all IDPS in the Northern region have demonstrated remarkable resilience and post-traumatic growth immediately following the war, despite multiple displacements, losses, separations, and heart-wrenching experiences they had to endure during the conflict period [13]. Tamil families are known to be close-knit and tend to function as a unit, rather than individuals in the event of a traumatic experience. The religious and spiritual beliefs along with cultural practices followed in these communities can be considered as major supportive factors. Hence social support from family, friends, and community relationships and the sense of belonging appeared to be a vital protective factor which played an important role in their recovery.

Alcohol, tobacco, and substance use were reported low numbers (7.0%, n = 41). However, an Island-wide STEPS survey conducted by the WHO in 2015, reported that approximately one-fifth (22.3%) of the younger age group 18–29 are estimated to be current smokers, and according to the National Youth Health Survey conducted by the Family Health Bureau, Sri Lanka in 2015, the current alcohol prevalence among adolescents, both school going and non-school going, was 10.2% [38, 39].

During the project planning stages, medical officers working in the Vavuniya district, our CIE group members, schoolteachers and parents of adolescents informed the research team about the high use of substances by adolescents in the Vavuniya district and the related social and family problems. Whilst measures were taken to protect the privacy and confidentiality of the participants, our results suggest that substance use is under-reported. This has previously been identified as a major problem in adolescents research [40, 41].

The present study also found that adolescents who dropped out of school were more likely to have depression and used at least one substance during their lifetime compared to adolescents who did not. These results agree with a study conducted by Gore (2001), which found that “young people who drop out of high school are more likely to be depressed than high school graduates” and Valkov (2018), states that “high school dropouts, have much higher rates of substance use compared to non-dropouts” [42, 43].

According to Rowan-Kenyon (2007), “Socioeconomic status is a well-known factor affecting dropout” [44]. This could explain the almost equal percentages of both males and females dropping out of school. The impact of poverty and the acceleration of inequality on adolescent mental health and school dropout have been reported globally [45–47]. Due to the prevailing extreme poverty in the rural areas of North, North Central, North Western and Eastern Provinces of Sri Lanka, many adult parents leave for foreign employment opportunities, leaving behind their children with grandparents or other family members [48, 49]. This could lead to negative impacts such as child neglect, mental health problems, poor school performance and dropping out, teenage pregnancies and substance use [50–53].

Dropping out of school, depression, hopelessness, and substance use constitutes small percentages in the study. Substance use itself seems to be underreported, as participants may not have been willing to share information. Adolescents’ reluctance to participate and provide correct answers in research has been reported as a major concern [54–56].

## Conclusions

The low prevalence of hopelessness and depression highlights the fact that these adolescents appear to be resilient in the face of adversity. Furthermore, significant negative correlations between hopelessness and depression with perceived social support and resilience suggest that social support and resilience could be protective factors against mental health issues in these adolescents. However, the prevalence of school dropouts calls for a focus on academic attainment to promote better educational outcomes in the adolescents of this conflict-affected region.

## Data Availability

Collected data is stored in a password-protected hard drive at the Institute for Research and Development, Sri Lanka. Individual Researchers/Organizations who are interested in using this data, can make an official request through info@ird.lk and obtain the data.

## Abbreviations

DSDs: Divisional Secretariat Divisions
ERC: Ethics Review Committee
GNDs: Grama Niladhari Divisions
IDPs: Internally Displaced Persons
CIE: Community Involvement and Engagement
RAs: Research Assistants
WHO: World Health Organisation
